# The development and initial validation of a new working time scale for full-time workers with non-standard schedules

**DOI:** 10.1186/s12889-022-13963-7

**Published:** 2022-08-20

**Authors:** Jennifer M. Cavallari, Rick Laguerre, Jacqueline M. Ferguson, Jennifer L. Garza, Adekemi O. Suleiman, Caitlin Mc Pherran Lombardi, Janet L. Barnes-Farrell, Alicia G. Dugan

**Affiliations:** 1 Department of Public Health Sciences, UConn School of Medicine, 263 Farmington Ave MC6325, Farmington, CT 06030-6325 USA; 2 Department of Medicine, Division of Occupational and Environmental Medicine, UConn School of Medicine, Farmington, USA; 3 Department of Psychological Sciences, Division of Industrial and Organizational Psychology, University of Connecticut, Mansfield, USA; 4 Department of Psychology, Division of Industrial and Organizational Psychology, Montclair State University, Montclair, USA; 5 Department of Epidemiology and Population Health, Stanford University, Stanford, USA; 6 Department of Human Development and Family Sciences, University of Connecticut, Mansfield, USA

**Keywords:** Shift work, Irregular shift system, Extended operation, Night work, Scale, Reliability, Validity, Work hours

## Abstract

**Background:**

Working time characteristics have been used to link work schedule features to health impairment; however, extant working time exposure assessments are narrow in scope. Prominent working time frameworks suggest that a broad range of schedule features should be assessed to best capture non-standard schedules. The purpose of this study was to develop a multi-dimensional scale that assesses working time exposures and test its reliability and validity for full-time workers with non-standard schedules.

**Methods:**

A cross-sectional study was conducted using full-time, blue-collar worker population samples from three industries - transportation (*n* = 174), corrections (*n* = 112), and manufacturing (*n* = 99). Using a multi-phased approach including the review of scientific literature and input from an advisory panel of experts, the WorkTime Scale (WTS) was created and included multiple domains to characterize working time (length, time of day, intensity, control, predictability, and free time). Self-report surveys were distributed to workers at their workplace during company time. Following a comprehensive scale development procedure (Phase 1), exploratory factor analysis (EFA) (Phase 2) and, confirmatory factor analysis (CFA) (Phase 3; bivariate correlations were used to identify the core components of the WTS and assess the reliability and validity (Phase 4) in three samples.

**Results:**

Phase 1 resulted in a preliminary set of 21 items that served as the basis for the quantitative analysis of the WTS. Phase 2 used EFA to yield a 14-item WTS measure with two subscales (“Extended and Irregular Work Days (EIWD)” and “Lack of Control (LOC)”). Phase 3 used CFA to confirm the factor structure of the WTS, and its subscales demonstrated good internal consistency: alpha coefficients were 0.88 for the EIWD factor and 0.76–0.81 for the LOC factor. Phase 4 used bivariate correlations to substantiate convergent, discriminant, and criterion (predictive) validities.

**Conclusions:**

The 14-item WTS with good reliability and validity is an effective tool for assessing working time exposures in a variety of full-time jobs with non-standard schedules.

**Supplementary Information:**

The online version contains supplementary material available at 10.1186/s12889-022-13963-7.

## Background

The impact of globalization and the increasing demand for 24/7 workers has been a cornerstone issue for epidemiologists, occupational health psychologists, and policy-makers for some time [1–3]. Working non-standard schedules, defined as work outside of the traditional 9 AM to 5 PM, Monday through Friday pattern, impacts work (e.g., job behavior and job attitudes), health (e.g., physical and mental health and health behaviors) as well as quality of life (e.g., work-family conflict, divorce) [4]. As the workplace becomes increasing complex through developments in organizational design, technological advances, and work arrangements [1], scholars are paying closer attention to work schedule factors that extend beyond non-traditional work hours, such as mandatory overtime [5, 6] and the irregularity of shifts [7], suggesting a greater need to accurately evaluate the nature and structure of schedules. Since the circadian disruption and resulting health consequences of night work are well established [8], shift irregularity is gaining attention due to its compounding nature. For example, schedule irregularity not only disrupts sleep, but it can have an additional negative effect on recovery and social life, which would not be fully captured by assessing night work alone. While initiatives like the European Working Time provide rights to workers through limits on weekly working hours, provisions for adequate breaks across workdays, and weeks as well as adding extra protections during night work, this is not the case for the United States where the Fair Labor Standards act provides provisions for overtime pay, yet does not limit to the amount of hours an employee can work in a week nor require employers to give breaks to their employees.

Working time can be characterized according to a series of domains that include 1) length; 2) time of day; 3) intensity; as well as social aspects of working hours which include 4) control; 5) predictability; 6) free time and 7) variability of working time [7]. This characterization is based upon the known biological mechanisms by which working time impacts health and well-being through physiological, behavioral, and psychosocial mechanisms [8, 9]. Working time impacts include fatigue, and disruption of circadian rhythms, sleep, and social schedules. Working time schedule characteristics can often have numerous health impacts with complex relationships. For example, shift work has been linked to both circadian misalignment with evidence of disturbed sleep impacts both independently as well through the pathway of circadian disruption [10]. Measurement of working time variables may be performed through quantitative and/or qualitative methods. Administrative databases from human resource applications may provide detailed quantitative data on some aspects of working time such as length, time of day, intensity, free time, and variability, but may not fully capture the social aspects of working time within the domains of control or predictability, such as when a worker is on call or had to come to work unexpectedly [7, 11, 12]. Surveys allow for subjective assessment of working time [12], but their use and applicability depend on the quality of their development and length, with shorter measures that prevent survey fatigue more desirable. Overall, there is no gold standard.

Typically, working time scales are unidimensional constructs that assess one aspect of schedules. An advantage of focusing on one schedule feature is that the measure will be short, but as a result, it sacrifices capturing nuances about a worker’s time—which may account for more variability in outcomes. Measures of working time can vary from each other in several ways. They may focus exclusively on the length and frequency of overtime [13], for example, but do not assess whether that overtime interfered with a person’s ability to have a personal life outside of work or when the overtime occurs [14]. Or, they may focus on an employee’s satisfaction with their schedule; but in return, they fail to capture whether the satisfaction has to do with a specific time of day [15]. The unique characteristics of essential service jobs (e.g., health care, corrections, transportation), where in the United States extended and rotating shifts are the norm and the prospect of working mandatory double shifts without advance notice is a foregone conclusion, suggests that a unidimensional measure of working time will consistently fall short of quantifying these workers’ exposures. To date, no comprehensive working time measure exists for workers, necessitating the need for a context-specific scale that evaluates multiple dimensions of work [5].

Therefore, the primary goal of this study is to identify survey items that fully describe working time characteristics, develop a parsimonious working time assessment scale, and test its reliability and validity for workers that are exposed to a variety of working time exposures with respect to length, time of day, intensity as well as social aspects of working hours (control, predictability, free time and variability). We choose to focus on three populations of workers –transportation workers, correctional officers, and manufacturing workers – due to their exposure to a variety of working time characteristics [16] as well as to increase the generalizability of our results. Our goal was to create a work time scale: 1) using a psychometrically reputable procedure; 2) that is able to predict quality of life outcomes; and 3) that is appropriate for full-time workers with non-standard schedules.

## Methods

### Study design

The WorkTime study is a cross-sectional, mixed methods study of workers examining the associations between working time characteristics and worker and family health and well-being. The current analysis focuses on the multiphase development of a working time scale using three study populations within the WorkTime cohort.

### Study populations

All three populations within the WorkTime cohort work within a New England State either within the state Department of Transportation (DOT), state Department of Corrections (DOC), or a privately owned manufacturing company. While the three populations are distinct in job titles and functions, they are similar with regard to numerous factors. All workers were employed full-time and had access to full medical benefits. The DOT and DOC workers were unionized, state-employees. The manufacturing employees were not unionized, and worked for one medium sized light-manufacturing company. Participants in the manufacturing sample were a subset of a larger longitudinal study of manufacturing workers at six small to medium companies. All surveys were completed with company approval while workers were on work time. Study protocols were reviewed and approved by the UConn Health Center’s Institutional Review Board. Signed informed consent was obtained by all study participants.

#### Sample population 1

Sample population 1 included Department of Transportation (DOT) workers. Transportation employees (including maintainers, crew leaders and supervisors) were recruited to take the survey at the beginning of their shift prior to a training at the regional transportation maintenance garages where they were stationed. Maintainers repair and maintain state roads by plowing, paving, grass-cutting and related work. A total of 232 employees were invited to complete a survey about their attitudes and experiences in work and life domains either at the beginning or end of their shift. Out of the total, 174 participants (75%) ranging in age from 22 to 62 years (Mean = 44.9, SD = 10.4) completed the survey and provided enough useable data for the analyses. The sample was primarily male (95%), white (69%), and reported working in the transportation industry an average of 10 years (SD = 10.1) (Table 1).

**Table 1:** Sample population demographics

	Sample 1		Sample 2		Sample 3	
	DOT (***n*** = 174)		DOC (***n*** = 112)		MFG (***n*** = 99)	
Characteristics	N *(%)*	M (SD)	N *(%)*	M (SD)	N *(%)*	M (SD)
**Age**		44.9 (10.4)		42.4 (6.5)		48.9 (12.2)
22–29	17 (10.1)		0		6 (6.1)	
30–39	34 (20.2)		44 (40.4)		15 (15.1)	
40–49	48 (28.6)		49 (45.0)		21 (21.2)	
50–59	63 (37.5)		16 (14.7)		26 (26.3)	
60+	6 (3.6)		0		20 (20.2)	
	*6 missing*		*3 missing*		*11 missing*	
**Tenure**		10.3 (10.1)		15.2 (5.2)		15.8 (10.1)
< 1–10	108 (62.8)		14 (12.5)		27 (27.3)	
11–20	30 (17.4)		85 (75.9)		27 (27.3)	
21–30	23 (13.4)		13 (11.6)		29 (29.3)	
31 or more	11 (6.4)		0		5 (5.1)	
	*2 missing*		*0 missing*		*11 missing*	
**Gender**						
Male	165 (94.8)		88 (78.6)		49 (49.5)	
Female	5 (2.9)		23 (20.5)		47 (47.5)	
	*4 missing*		*1 missing*		*3 missing*	
**Race / Ethnicity**						
White, European Descent	120 (69.0)		67 (59.8)		65 (65.7)	
Black, African American, African	22 (12.6)		22 (19.6)		9 (9.1)	
American Indian, Alaska Native	1 (0.6)		1 (0.9)		2 (2.0)	
Other Race or Multiracial	22 (12.6)		18 (16.1)		19 (19.2)	
	*9 missing*		*4 missing*		*4 missing*	
**Education**						
Less than high school	1 (0.6)		0		2 (2.0)	
High school graduate	98 (56.3)		20 (17.9)		22 (22.2)	
Graduate Equivalency Degree (GED)	11 (6.3)		2 (1.8)		4 (4.0)	
Some college, technical school, or certification program	46 (26.4)		41 (36.6)		23 (23.2)	
College degree	15 (8.6)		49 (45.0)		42 (29.1)	
	*3 missing*		*0 missing*		*6 missing*	
**Job Title**						
Supervisor	6 (3.5)		112 (100)		21 (21.2)	
Non-supervisor	164 (96.4)		0		68 (68.7)	
	*4 missing*		*0 missing*		*10 missing*	
**Family Income**						
$10,000 - $24,999	1 (0.6)		0		9 (9.0)	
$25,000 - $49,999	15 (8.6)		0		16 (16.2)	
$50,000 - $74,999	51 (29.3)		5 (4.5)		14 (14.1)	
$75,000 - $99,999	37 (21.3)		23 (20.5)		15 (15.2)	
$100,000 - $149,999	45 (25.9)		50 (44.6)		20 (20.2)	
More than $150,000	15 (8.6)		27 (30.4)		14 (14.1)	
	*10 missing*		*0 missing*		*11 missing*	
**Financial Situation**						
Able to live comfortably	53 (30.5)		31 (27.7)		38 (38.4)	
Meet basic expenses with a little left over for extras	61 (35.1)		56 (50.0)		29 (29.3)	
Just meet basic expenses	44 (25.3)		22 (19.6)		27 (27.3)	
Don’t even have enough to meet basic expenses	8 (4.6)		3 (2.7)		2 (2.0)	
	*8 missing*		*0 missing*		*3 missing*	
**Marital Status**						
Married or live with a partner	109 (62.6)		88 (78.6)		65 (65.7)	
Widowed	3 (1.7)		0		2 (2.0)	
Divorced or separated	26 (14.9)		13 (11.6)		13 (13.1)	
Single, never married	31 (17.8)		11 (9.8)		17 (17.2)	
	*5 missing*		*0 missing*		*2 missing*	
**Works Other Jobs**						
Yes	69 (39.7)		27 (24.1)		12 (12.1)	
No	101 (58.0)		85 (75.9)		82 (82.8)	
	*4 missing*		*0 missing*		*5 missing*	

*DOT* Department of Transportation, *DOC* Department of Corrections, *MFG* Manufacturing

#### Sample population 2

Sample population 2 included Department of Corrections (DOC) supervisors. Correctional supervisors (including lieutenants, captains, counselor supervisors, deputy wardens, and parole managers) were recruited to take the survey during an off-site mental health training. Correctional supervisors work within the state prisons (or jails) supervising correctional officers. A total of 137 full-time employees were invited to complete a survey about their attitudes and experiences in work and life domains during a professional development mental health training day. Out of the total, 112 participants (82%) ranging in age from 33 to 58 years (Mean = 42.4, SD = 6.5) completed the survey and provided enough useable data for the analyses. The sample was primarily male (79%), white (60%), and had worked in corrections an average of 15 years (SD = 5.2) (Table 1).

#### Sample population 3

Sample population 3 included manufacturing workers within a single manufacturing company. Manufacturing workers were recruited to take the survey during their workday. All manufacturing workers on site were considered eligible and invited to participate in the study; no exclusion criteria were specified. Employees of all job classifications participated (e.g., production, sales, administrative, managerial staff). A total of 290 workers were invited to complete a survey about their attitudes and experiences in work and life domains. Out of the total, 99 responded (34%) to the survey and provided enough useable data for the analyses. Half of sample was male and they were primarily white (66%), ranged in age from 22 to 74 years (Mean = 48.9, SD = 12.2), and they reported working at their company an average of 15.8 years (SD = 10.1) (Table 1).

### Scale development and validation

The WorkTime Scale (WTS) development proceeded over four phases. In phase one, we identified items of working time from the extant literature and synthesized research, as well as feedback from subject-matter experts and focus groups of workers [16] from within the WorkTime project. During the second phase of the study, we employed a systematic scale development procedure to reduce the number of WTS items in a sample of transportation workers. Using correction officers and manufacturing workers, phase three confirmed the psychometric properties (e.g., reliability) of the WTS, and phase four validated the WTS using bivariate correlations with other measures.

#### Phase 1: worktime scale (WTS) development

The working time construct was categorized based on the Härmä et al. framework—length, time of day, intensity, and social aspects of working [7]. The 21-item WTS was compiled based on a review of existing surveys assessing working time. We considered prominent surveys employed in the United States including the National Health Interview Survey [17], the Quality of Worklife Questionnaire [18], the American Time Use Survey [19] and the Employment Instability, Family Well-being and Social Policy Network (EINet) measures for Precarious Work Schedules [20] as well as the European Working Conditions Survey [21]. Each survey was reviewed to identify relevant measures within the working time framework and considered to ensure that we developed an exhaustive preliminary version of the WTS. Further, we considered items that have been previously utilized by researchers at the Center for Promotion of Health in the New England Workplace (CPH-NEW) [22] and the UConn Study of Aging and Musculoskeletal Disorders [23]. Lastly, we sought input from subject-matter experts in epidemiology and occupational health psychology, to iteratively revise and improve the WTS items until reaching agreement.

Using fundamental concepts within exposure assessment, we aimed to characterize working time exposures by their frequency, duration, and intensity. Given that certain working time constructs touched on duration (e.g. length of work shift) and intensity (time between shifts), we opted to assess the frequency by which poor working time exposures occurred. Thus, the original items of the WTS were developed with items assessed based on the frequency of occurrence on a Likert Scale ranging from 1 (Always) to 5 (Never). Furthermore, since the goal of the WTS was to link working time exposures with health outcomes (including psychosocial effects as well as mental and physical health impacts), we assessed working times exposures across all jobs and over the course of a year. The preliminary version of the WTS asked respondents “Thinking about all jobs that you work, and including all overtime, say how often the following occurred over the LAST YEAR.” Respondents selected options on a 5-point Likert scale (1 = always, 2 = usually, 3 = sometimes, 4 = rarely, 5 = never). The majority of items were reverse coded so that high WTS ratings can be interpreted as having higher exposures to poor work schedule characteristics. The Likert scale has been reversed in Tables to ease interpretation.

The original WTS were developed for six dimensions (length, time of day, intensity, control, predictability and free time) with each item being assessed based on the frequency of occurrence on a Likert Scale ranging from 1 (Always) to 5 (Never). However, the construct of variability was assessed using a single item asking “What best describes your usual schedule/primary shift (excluding overtime)?” with five response items including 1 = fixed; 2 = rotating days; 3 = rotating hours; 4 = rotating days and hours; and 5 = no pattern. These shift options were meant to capture variability in both the days of week worked as well as the start/stop time. Since the response options for the variability scale was categorical rather than continuous, it could not be factored into the WTS.

#### Confirmation survey measures

To confirm the WTS items, we compared the items against two types of validated survey measures. The first group included 4 schedule-related measures, and the second group included 4 psychosocial- and sleep-related outcomes.

With respect to schedule-related measures, a single-item assessment of primary job overtime was adapted from Cully [13]. Respondents were asked “How many overtime HOURS did you work at this job in the last month (include paid and unpaid overtime work)?” A numerical value was provided to indicate the number of overtime hours worked in the past month. We adapted three single-item measures from Lambert and Henly [14] to assess non-standard work schedules: 1) a measure of working time irregularity that asked “What best describes your usual schedule/primary shift (excluding overtime)?” on a 5-point scale, where 1 = “Fixed (you usually work the same days of the week and start around the same time each day)” and 5 = “There is no pattern to my work schedule.”; 2) a measure of working time control/flexibility that asked respondents to evaluate whether “It is difficult to take time off from work to take care of personal or family matters” on a 5-point Likert scale (1 = strongly disagree, 5 = strongly agree); and 3) a measure of working time predictability that asked respondents “How far in advance do you usually know what days and hours you will need to work?” on a 7-point scale, where 1 = one day or less in advance and 7 = my schedule never changes.

With respect to psychosocial and sleep outcomes, depression was assessed using the 8-item revised Center for Epidemiologic Studies Depression (CES-D) Scale [24]. Respondents were provided with the stem “Below is a list of some of the ways you may have felt. Please indicate how often you have felt this way during the PAST WEEK.” A sample item was “I am depressed” and response options were rated on a 4-point scale, where 1 = “rarely or none of the time (less than 1 day per week)” and 4 = “all of the time (5-7 days per week).” Sleep duration was assessed using a single-item measure of total sleep from the Pittsburgh Sleep Quality Index (PSQI) [25]. The item was “In the PAST MONTH, about how many hours of sleep did you typically get per 24-hour period during the WORK WEEK?” and response options were on a 12-point scale from 0 hours to > 10 hours. Two types of job demands were assessed with the Job Content Questionnaire (JCQ) [26]. A 4-item subscale for psychological job demands (sample item “My job requires working very fast”) and a 4-item subscale for physical job demands (sample item “I am often required to move or lift very heavy loads on my job”) were used, and response options were on a 4-point scale (1 = strongly disagree, 4 = strongly agree).

#### Phase 2: initial item reduction and exploratory factor analysis (EFA)

The purpose of phase 2 was to determine whether the theorized items, created in phase 1, mapped on to their respective domains. During this phase, the factor structure and initial psychometric characteristics of the WTS were assessed using exploratory factor analysis (EFA). We used Hinkin’s scale development procedure because it is a highly reputable approach for designing measures for use in organizational research [27]. We used sample population 1 (transportation workers) to delete problematic items and conduct an exploratory factor analysis. First, we conducted scale inter-item correlations and dropped items that correlated lower than 0.40 with all other items, which should have similar associations with one another [28]. Next, an exploratory factor analysis (EFA) with maximum likelihood (ML) estimation was used on the remaining items to determine the structure of the item set. A scree plot [29] and Kaiser criterion (eigenvalues > 1.0, [30]) were then used to determine the number of factors to retain. EFA was repeated with removal of additional items loading below 0.40 until an acceptable variance was achieved. Phase 2 data analysis was performed in SPSS (Version 25).

#### Phase 3: confirmatory factor analysis (CFA)

Following the EFA in phase 2, we attempted to replicate the factor structure of the WTS in two distinct samples (sample populations 2 and 3) using confirmatory factor analysis (CFA). Sample population 2 consisted of correctional supervisors and sample 3 consisted of manufacturing employees.

We used multiple indices to assess model fit [27]. Hu and Bentler [31] recommend reporting at least two fit indices and considering them in combination with one another. We reported the Comparative Fit Index (CFI), Tucker-Lewis Index (TLI), standardized root mean squared residual (SRMR), and root mean squared error of approximation (RMSEA). A good fit is evidenced by a CFI/TLI > 0.90, SRMR < 0.08, and a RMSEA < 0.08 [32]. However, researchers have cautioned against strict adherence to cutoffs for fit indices [32, 33]; therefore, we follow Jackson et al.’s [34] suggestion to interpret results with the factor loadings in mind. Thus, a model may still be acceptable if the fit indices are not ideal but the factor loadings are strong.

It is important to highlight that Hinkin [27] suggested that modification indices be used to improve model fit, and they should be reported. Modification indices recommend changes that researchers can make to account for the most variance in data, and this tool should be used in concert with theoretical and practical considerations [27]. Thus, several adjustments were made to the confirmatory factor analysis on the basis of modification indices. Specifically, error terms were correlated and an item was switched from one factor to another factor. Phase 3 analysis were performed in Mplus 8.1.

#### Phase 4: worktime scale (WTS) validation

The convergent validity of the WTS was evaluated by comparing responses of the WTS with other validated measures in sample populations 2 and 3. Specifically, convergent validity was assessed by ensuring that WTS is correlated with constructs that it is theoretically related to, including other schedule-related measures as well as psychosocial and sleep outcomes. Specifically, given the literature on working long and irregular hours, we expected that the WTS would be positively associated with depression and appraisals of engaging in more demanding work while it would be negatively associated with sleep duration. Evidence for discriminant validity was generated by assessing whether the WTS exhibited associations with outcomes in the expected direction (e.g., higher EIWD should be related to lower levels of sleep), and whether the WTS differentiated between respondents’ appraisals of psychological and physical job demands.

## Results

### Phase 1: worktime scale (WTS) development

Phase 1 item development resulted in a 21-item WTS representing working time constructs including length (3 items), time of day (4 items), intensity (3 items), control (3 items), predictability (4 items) and free time (4 items) (Table 2).

**Table 2:** Summary of the WorkTime Scale survey items and domain classifications by population. Respondents assessed the frequency of each working time exposure over the last year for all jobs held on a Likert Scale: Never (1), Rarely (2), Sometimes (3), Usually (4) and Always (5). Except where noted, higher values indicate more frequent exposure to poor working time characteristics

Items		Domain	DOT (***n*** = 174)	DOC (***n*** = 114)	MFG (***n*** = 99)
			M (SD)	M (SD)	M (SD)
Q1	I worked more than 12 hours per day	Length	3.0 (0.9)	2.8 (0.9)	1.5 (0.8)
Q2	I worked more than 48 hours per week	Length	3.2 (1.0)	3.3 (1.1)	2.0 (1.2)
Q3	I worked overtime	Length	3.6 (0.9)	3.2 (1.1)	2.8 (1.2)
Q4	I worked some early morning hours between 5 am and 8 am	Time of day	3.2 (1.1)	3.4 (1.2)	2.8 (1.4)
Q5	I worked at least 3 daytime hours between 8 am and 6 pm^a^	Time of day	1.8 (1.1)	2.0 (1.2)	1.8 (1.4)
Q6	I worked at least 3 evening hours after 6 pm	Time of day	3.3 (1.1)	3.2 (1.3)	2.0 (1.3)
Q7	I worked at least 3 overnight hours between 11 pm and 5 am	Time of day	3.1 (1.1)	2.6 (1.4)	1.3 (0.6)
Q8	I worked 6 or more days in a row	Intensity	3.3 (1.0)	2.8 (1.2)	2.0 (1.1)
Q9	I had two or more days off in a row^a^	Intensity	3.1 (0.8)	2.1 (0.9)	2.7 (1.4)
Q10	I had less than 11 hours between shifts	Intensity	3.1 (0.9)	3.0 (1.1)	1.8 (1.3)
Q11	I was on call (expected to immediately provide work or service if contacted or called)	Control	3.5 (1.1)	2.4 (1.4)	1.7 (1.2)
Q12	I worked mandatory overtime	Control	3.8 (1.0)	2.1 (1.1)	1.7 (1.1)
Q13	I had control over my work schedule^a^	Control	3.7 (1.1)	2.6 (1.3)	2.6 (1.4)
Q14	I had to go to work unexpectedly at times when I was not scheduled to work	Predictability	3.3 (1.0)	2.0 (1.0)	1.7 (1.0)
Q15	I unexpectedly had to work more than an hour later than I was scheduled to work	Predictability	3.2 (0.9)	2.5 (0.9)	2.2 (0.9)
Q16	I knew my schedule in advance^a^	Predictability	2.8 (1.1)	1.5 (0.7)	1.8 (1.1)
Q17	Last minute adjustments were made to my schedule	Predictability	3.0 (1.0)	2.1 (1.0)	1.9 (0.9)
Q18	I worked on a Sunday	Free time	3.2 (1.1)	3.1 (1.4)	1.8 (1.2)
Q19	I worked on the weekend	Free time	3.4 (1.0)	3.3 (1.2)	2.3 (1.2)
Q20	I worked on a holiday	Free time	3.1 (1.0)	3.1 (1.4)	1.7 (1.1)
Q21	I worked during a special event (e.g., birthday party, wedding, graduation party, etc.)	Free time	2.8 (1.1)	2.9 (1.3)	1.8 (1.1)

^a^Items were reverse coded, so higher values indicate exposure to poor working time conditions

*DOT* Department of Transportation, *DOC* Department of Corrections, *MFG* Manufacturing

### Phase 2: initial item reduction and exploratory factor analysis (EFA)

#### Sample population 1

With respect to their working time exposures, DOT workers reported high frequency of poor working time exposures including *overtime (Q3)*, *on-call (Q11)*, *mandatory overtime (Q12)*, and *low schedule control (Q13)*, with each item having a mean of 3.5 or higher equating to a frequency between sometimes (3) and usually (4) (Table 2). In fact, the majority of working time exposure items had mean scores of 3 or more with the exception of *daytime hours (Q5)*, *advance schedule notice (Q16)*, *special event (Q21)*.

#### Initial item reduction and exploratory factor analysis (EFA)

As a results of the first-round EFA, three items were dropped (*2 or more days off (Q9)* from the intensity domain, *low schedule control (Q13)* from the control domain, and *advance schedule notice (Q16)* from the predictability domain). The results of the second-round EFA suggested a three-factor structure and three additional items (*daytime hours (Q5)* from the time of day domain, *quick turnover (Q10)* from the intensity domain, and *special event (Q21)* from the free time domain) had loadings that were below 0.40, and were subsequently dropped.

Our thirdround EFA resulted in the removal of one additional item for having a loading below 0.40 (*last minute schedule adjustments (Q17)* from the predictability domain). This third-round EFA yielded the best solution, a 14-item two-factor structure which accounted for 64.8% of the total variance in the items (Table 3), which is above the 60% threshold for a sound scale [27]. The first factor pertained to extended and irregular work days (EIWD) and consisted of nine items (coefficient alpha = 0.95). The second factor represented a lack of control (LOC) and contained five items (coefficient alpha = 0.87). The correlation between these two factors was 0.71. See Table 3 for the factor loadings of the EFA results, and see Appendix Table A1 for the model building tests for the EFA, which demonstrate that the 2-factor structure has the best fit *and* meets the Kaiser criterion (eigenvalues > 1.0).

**Table 3:** Factor structure of WorkTime Scale survey items in 3 blue-collar worker samples

Items		Factor Loadings					
		DOT (***n*** = 174)		DOC (***n*** = 114)		MFG (***n*** = 99)	
		EIWD	LOC	EIWD	LOC	EIWD	LOC
Q1	I worked more than 12 hours per day	0.75		0.63		0.70	
Q2	I worked more than 48 hours per week	0.83		0.69		0.75	
Q3	I worked overtime		0.53	0.58		0.58	
Q4	I worked some early morning hours between 5 am and 8 am	0.84		0.07 ns		0.24	
Q5	I worked at least 3 daytime hours between 8 am and 6 pm	–	–	–	–	–	–
Q6	I worked at least 3 evening hours after 6 pm	0.81		0.64		0.72	
Q7	I worked at least 3 overnight hours between 11 pm and 5 am	0.80		0.45		0.52	
Q8	I worked 6 or more days in a row	0.80		0.77		0.77	
Q9	I had two or more days off in a row	–	–	–	–	–	–
Q10	I had less than 11 hours between shifts	–	–	–	–	–	–
Q11	I was on call (expected to immediately provide work or service if contacted or called)		0.61		0.67		0.85
Q12	I worked mandatory overtime		0.58		0.54		0.65
Q13	I had control over my work schedule	–	–	–	–	–	–
Q14	I had to go to work unexpectedly at times when I was not scheduled to work		0.95		0.85		0.77
Q15	I unexpectedly had to work more than an hour later than I was scheduled to work		0.60		0.71		0.73
Q16	I knew my schedule in advance	–	–	–	–	–	–
Q17	Last minute adjustments were made to my schedule	–	–	–	–	–	–
Q18	I worked on a Sunday	0.88		0.86		0.86	
Q19	I worked on the weekend	0.87		0.89		0.85	
Q20	I worked on a holiday	0.81		0.94		0.82	
Q21	I worked during a special event (e.g., birthday party, wedding, graduation party, etc.)	–	–	–	–	–	–
Cronbach’s Alpha Coefficient		0.95	0.87	0.88	0.76	0.88	0.81

*DOT* Department of Transportation, *DOC* Department of Corrections, *MFG* Manufacturing, *EIWD* Extended and Irregular Workdays, *LOC* Lack of Control, *ns* Not significant

### Phase 3: confirmatory factor analysis (CFA)

#### Sample populations 2 and 3

In terms of harmful working time exposures, DOC supervisors within sample population 2 reported higher frequency with means of over 3 (sometimes) for the following working time exposures: *48 or more hours weekly (Q2), overtime (Q3), early morning hours (Q4), evening hours (Q6), quick turnovers (Q10), Sunday (Q18), weekend (Q19)* and *holiday (Q20)* (Table 2). *Daytime hours (Q5), unexpected call-in (Q14),* and *advance schedule notice (Q16)* were on average less frequent with means of 2 or below indicating occurring rarely (2) or never (1). In terms of working time exposures, for the sample population 3 of manufacturing workers as a whole, no means were above 3 (sometimes) although both *overtime (Q3)* and *early morning hours (Q4)* had the highest frequency of harmful working time exposures with a mean of 2.8 (Table 2).

#### Confirmatory factor analysis

We were able to replicate the majority of the EFA results from sample population 1 in a CFA conducted on sample population 2. However, based on the suggestions of Hinkin [27], we leveraged the modification indices in sample population 2 to improve model fit and further refine the 14-item WTS. Specifically, within the CFA for sample population 2, error (or unexplained) variances were allowed to covary between six item pairs: *12 or more hours daily (Q1) and 48 hours or more weekly (Q2)*, both from the length domain; *48 or more hours weekly (Q2) and overtime (Q3)*, both from the length domain*; 12 or more hours daily (Q1) and overtime (Q3),* both from the length domain; *early morning hours (Q4) and overnight hours (Q7)*, both from the time of day domain*; 6 or more days on (Q8) and holiday (Q20),* from the intensity and free time domains, respectively; *Sunday (Q18) and holiday (Q20)*, both from the free time domain - these item pairings essentially mean that there was more overlap (or higher interrelatedness) between these specific working time features than the CFA could capture*.*

Another modification pertained to cross-loading *overtime (Q3)* so that it was an indicator for both factors (EIWD and LOC). Once this change was made, *overtime (Q3)* lost significance as an indicator on the LOC factor, and the decision was made to delete the non-significant path. This ultimately resulted in the *overtime (Q3)* item being exclusively on the EIWD factor. The WTS, therefore, distinguished between overall *overtime* (*Q3*) from the length domain and *mandatory overtime* (*Q12*) from the control domain by having these items load on different factors (EIWD and LOC, respectively). The final two-factor model had adequate fit, with a chi-square value of 148.76 (df = 70; *p* < 0.001), a CFI/TLI of 0.91/0.89, an SRMR of 0.08, and an RMSEA of 0.10 (see Appendix Table A2). With the exception of *early morning hours* (*Q4)* (from the length domain), the factor loadings ranged from 0.45 to 0.94 (Table 3). Due to sample population 2 consisting of corrections supervisors who generally had control over early morning schedules, we determined that the *early morning hours (Q4)* loading accurately represented this group but also acknowledged that this item would appropriately capture non-supervisory staff’s shifts. In all, the final WTS is a measure that has 10 items for the EIWD factor and 4 items for LOC factor (Tables 3, A4). The Pearson correlation coefficient for these two factors was 0.26. The WTS had good internal consistency: Cronbach’s alpha coefficients were 0.88 for EIWD and 0.76 for LOC (Table 3).

The CFA in sample population 2 was replicated in sample population 3. We fit the final two-factor model for the WTS (i.e., 10-item EIWD, and 4-item LOC) and used identical information to assess model fit. Based on the modification indices and following a similar rationale for sample population 2, we allowed error variances to covary, with some of them overlapping with those observed in sample population 2. The six item pairs that covaried were: *Sunday (Q18)* and *holiday (Q20),* both from the free time domain*; 48 or more hours weekly* (*Q2)* and *Sunday* (*Q18),* from the length and free time domains, respectively; *6 or more days (Q8)* and *holiday (Q20),* from the intensity and free time domains, respectively; *12 or more hours daily (Q1)* and *48 or more hours weekly* (*Q2),* both from the length domain; *48 or more hours weekly (Q2)* and *6 or more days (Q8),* from the length and intensity domains, respectively; *on-call* (*Q11)* and *Sunday (Q18)*, from the control and free time domains, respectively. The two-factor model had an adequate fit to the data with a chi-square value of 136.32 (df = 70; *p* < .001), a CFI/TLI of 0.91/0.88, an SRMR of 0.07, and an RMSEA of 0.10 (see Appendix Table A3). As seen in Table 3, factor loadings ranged from 0.24 to 0.86, and Cronbach’s coefficient alphas were also good for EIWD (0.88) and LOC (0.81).

### Phase 4: worktime scale (WTS) validation

#### Convergent Validity

With respect to schedule related measures, the results indicated significant correlations (0.28–0.43) between the WTS and a measure of primary job overtime (Table 4), suggesting that the WTS was evaluating a similar construct measured by the primary job overtime assessment and had good convergent validity. Additionally, there were significant correlations between the WTS and the measure of precarious work schedules, further suggesting good convergent validity of the WTS (Table 4). In summary, people who rated high on dimensions of the WTS reported working more overtime hours in the past month, tended to describe their schedules as non-fixed, reported greater difficulty in taking time off work for personal or family matters, and reported less advanced notice for schedule assignments.

**Table 4:** Correlations between the Work Time Scale and other schedule-related measures

		DOC (***n*** = 114)		MFG (***n*** = 99)	
Measure	Sample Item(s)	EIWD	LOC	EIWD	LOC
Primary Job Overtime	How many overtime HOURS did you work at this job last month (include paid and unpaid overtime work)?	0.28^**^	0.34^**^	0.43^**^	0.36^**^
Precarious Work Schedules	What best describes your usual schedule/primary shift (excluding overtime)?	0.38^**^	−0.05	−0.14	−0.13
	It is difficult to take time off from work to take care of personal or family matters.	0.11	0.25^**^	0.21	0.27^*^
	How far in advance do you usually know what days and hours you will need to work?	−0.19^*^	− 0.22^*^	− 0.11	− 0.28^*^

*P < 0.05; **P < 0.01

*DOC* Department of Corrections, *MFG* Manufacturing, *EIWD* Extended and Irregular Workdays, *LOC* Lack of Control

#### Criterion validity

The two factors of the WTS were significantly correlated with depression, total sleep, and job demand appraisals (Table 5). Collectively, this provides evidence for the use of the WTS as an exposure measure for outcomes important to workers.

**Table 5:** Correlations of the Work Time Scale with psychosocial and sleep outcomes

Measure	DOC (***n*** = 114)		MFG (***n*** = 99)	
	EIWD	LOC	EIWD	LOC
CES-D Scale	0.14	0.27^**^	0.18	0.18
PSQI: Total Sleep	−0.25^**^	−0.05	−0.27^*^	− 0.20
JCQ: Psychological Demands	−0.07	0.27^**^	0.35^**^	0.34^**^
JCQ: Physical Demands	0.25^**^	0.09	0.07	0.09

*P < .05; **P < .01

*DOC* Department of Corrections, *MFG* Manufacturing, *EIWD* Extended and Irregular Workdays, *LOC* Lack of Control, *CES-D* Center for Epidemiologic Studies-Depression, *PSQI* Pittsburgh Sleep Quality Index, *JCQ* Job Content Questionnaire

#### Discriminant validity

Evidence of discriminant validity was observed because significant associations (e.g., − 0.25, *p* < 0.01) in the expected direction for total sleep were found for people who were high on the EIWD factor of the WTS, while no such association existed for the LOC aspect of the WTS (e.g., − 0.05) (Table 5). Further, EIWD and LOC differentiated between the types of job demands people experienced, and sample characteristics played a role in the nature of associations observed. In the DOC sample, the two dimensions of the WTS had a low/moderate bivariate correlation of 0.26, which allowed the WTS to make greater distinctions between the physical and psychological aspects of work (Table 5). This is evidenced by the fact that the EIWD factor was significantly correlated with JCQ physical demands (0.25), and the LOC factor was significantly correlated with the JCQ psychological demands (0.27) in the DOC sample (Table 5)—suggesting that working long hours takes a physical toll on DOC workers, while lacking schedule control takes a greater mental toll. In the manufacturing sample, however, the WTS dimensions had a bivariate correlation of 0.73. This contributed to the finding that both WTS dimensions (i.e., EIWD and LOC) had significant associations with JCQ psychological demands and no associations with JCQ physical demands among manufacturing workers (Table 5).

Providing further support of the discriminant validity of the WTS, our model building CFA tests confirmed that the WTS is best operationalized as two distinct dimensions (EIWD and LOC) rather than a single unidimensional construct (see Appendix Table A2 and Appendix Table A3). Overall, there is initial evidence that the WTS discriminates between two aspects of non-standard schedules, EIWD and LOC, and these aspects seem to correlate differently depending on the population of interest. Moreover, the WTS exhibits significant associations with physical and psychological outcomes in the expected direction.

## Discussion

We developed a 14-item WorkTime scale (WTS) that characterized working time characteristics based on an established framework [7]. Within three distinct populations of full-time work forces, exploratory factor analysis identified two subscales including one reflective of extended and irregular work days (EIWD) and another reflective of lack of schedule control (LOC). The WTS demonstrated good convergent validity, showing significant correlations with both schedule-related measures as well as psychosocial and sleep outcomes.

Based on the Härmä et al. framework [7], we anticipated that the scale would load upon six dimensions, corresponding to schedule length, time of day, intensity, control, predictability, and free time. Yet, upon evaluation for the three populations within the study, the inherent inter-relatedness of these schedule factors revealed patterns that could be grouped as either EIWD or LOC. For example, if a worker exhibits a pattern of having extended or irregular work days (EIWD)– as evidenced by working more than 12 hours a day (Q1), occasionally working early morning hours (Q4), evening hours (Q6) and overnight hours (Q7) – they will experience worsened work and life outcomes. Moreover, if this same worker were frequently on call (Q11), had to work unexpectedly on their days off (Q14), and unexpectedly worked longer hours than scheduled (Q15), they would exhibit an overlapping yet differential set of adverse outcomes when compared to EIWD. The results of our initial validation of the WTS aligns with research demonstrating the interrelatedness of working time features in outcomes important for longevity at work [35]. We extend the literature on working time exposures by going beyond single-item assessments and evaluating patterns of exposures, which has its benefits. Specifically, due to its multi-item nature, the WTS captures more of the working time domains than other scales, which has the distinct advantage of yielding a scale with greater content validity and reliability than single-item working time exposure measures [36]. In practice, this means that the WTS will enable investigators to make stronger inferences about the adverse impact of working time characteristics, particularly when comparing different worker populations.

With the two factor composite measure, we were allowed to link the working time exposures with health and well-being outcomes among the three populations. In fact, schedule control has been shown to be an important predictor of poor self-related health [37], high schedule control or worker flexibility, is associated with better health and well-being [38]. Yet, low job control has been showing increasing trends in the US workforce from 2002 to 2014 [39]. The components of EIWD, which are long work hours and irregular work schedules, have also been independently linked to poor health outcomes. Long work hours have been linked to poor cardiovascular health, mental health, health behaviors, and sleep as well as increased fatigue and workplace accidents [40]. Irregular work hours have been linked to increased work-life conflict as well as poor cardiovascular and mental health [38].

The study is strengthened by the use of an established framework [7] to characterize working time across numerous dimensions. Furthermore, the study comprehensively captured working time exposures across all jobs, rather than just one job held by each individual, which in the current populations applied to 29% of survey respondents. In addition, three distinct populations were used to develop and validate the survey measure to expand the generalizability of the results.

Yet, the study is not without weakness. Several items were dropped in this study. For example, “I had two or more days off in a row (Q9)” was one item dropped. One reason for this may be that measuring the number of free days may not be the same as capturing the extent of EIWD. For example, a person can have a demanding and irregular schedule for the days they are actually at work, while still having free days off within a week. Moreover, another item dropped pertained to “I had control over my work schedule (Q13)”. Perhaps, the wording of this item was too vague to differentiate among people, and it is better to use more specific items that capture control of schedule features. This may be one explanation for why the LOC factor ended up with 4 items; they were all detailed (e.g. I unexpectedly had to work more than an hour later than I was scheduled to work) and could better differentiate among people’s lack of control at work.

Furthermore, survey measures of any exposure are prone to exposure misclassification, and more objective measures of working time, for example from payroll data are preferred. Often times, payroll data has shift start and stop times to calculate pay and overtime for the company and allow for the calculation of numerous aspects of working time including length, time of day, intensity, free time, and variability, and sometimes control, as is the case for hospital based systems that track desired and received shifts [7]. Yet, payroll data often lacks information on the social aspects of working time, especially predictability and control, and only accounts for one job, not all which can be better captured via a questionnaire. Likewise, payroll data is often difficult to obtain as companies may lack the ability to de-identify data and are reluctant to share what may be sensitive information with researchers. A thorough capture of working time exposures would include both payroll data along with survey items to fill in working time exposure such as predictability or control.

Prior studies examining the validity of self-reported survey items on work schedule characteristics show heterogeneity in exposure misclassification based on work schedule type, yet support a bias towards the null [12]. We asked workers to assess the frequency of working time exposures over the past year, as part of an epidemiological study. It is our intent to correlate these working time exposures with health outcomes where the mechanism of action is on the time scale of years rather than a month. However, given that we are asking people to assess exposures over a long period of time, the exposures are prone to recall bias and the actual report of exposure may be a reflection of more recent exposure rather than trends over the past year. This could either under- or over-estimate exposure depending on the variability of the working time exposures over the course of a year.

Future use of the WTS should include more varied worker populations with new survey items to assess additional facets of nonstandard work arrangements and work organizational factors in general. For this validation study we chose three different study populations to increase the generalizability of our results. While all were full-time employees with benefits, the populations differed with respect to job function as well as the distribution of age, gender, and family income. The healthy worker survivor bias may have played a role where workers more suited to the challenges of poor work time exposures more highly represented in the study populations [41]. Furthermore, the study populations were predominately male, white and married. The WTS factors and composite measure may look different among workforces that have higher percentages of females, non-white workers, single workers, as well as within industries with lower wages and fewer/no benefits.

Future scales should consider the incorporation of survey items to assess work schedule variability. Identifying whether workers work the same days each week, start and end at the same time each workday, or work the same number of hours every week would provide additional information on work schedule variability. An expanded scale along with the 14 items already identified could be informative for assessing working time exposures across a broader range of full-time workers.

The WTS can be used in combination with other work organizational factors to assess and ultimately improve the well-being of workers. In addition to working time characteristics, other salient working arrangement characteristics can impact the health and well-being of workers. All of the worker populations studied were full-time and permanent employees with access to health benefits. This is in contrast to contingent work which is characterized by less secure work arrangements with changes in employment based on employer demand [42] or precarious employment which is characterized by some degree of insecurity; temporariness; inadequate pay; vulnerability to unfair treatment; lack of ability to negotiate benefits, pay, work schedule, and leave; as well as the lack of a social safety net [43]. There is often overlap between workers characterized as contingent or precarious, with both groups experiencing non-standard work arrangements and possibly non-standard work schedules. Precarious employment has been linked to poor mental health [44–47], general health [46] and mortality [44]. There may be benefit in exploring the interaction between contingent and precarious work arrangements and working time factors as a driver of these adverse outcomes. The large majority (99.4%) of workers in this population reported a family income of over $25,000 which, depending on family size, correlates with living above the poverty guidelines. Evidence suggests that workers in lower socioeconomic positions are exposed to greater worker organizational hazards including job insecurity which plays a role in perpetuating occupational health disparities [48]. Future research should continue to examine the interaction between low socioeconomic position and worktime pay and working time factors. Overall, the WTS can be used alongside work characterizations including benefit status as well as contingent or precarious employment to further identify hazardous work schedule characteristics.

Furthermore, it should be noted that working time characteristics are highly contextual to the laws, practices and norms within industries, states, and countries. For example, overall Europeans work less hours per week and weeks per year than US Americans, namely 14% fewer hours were documented within the period of 1983–2015 [49]. With respect to the WTS, the survey items are broad enough to capture working time characteristics across a wide range of industries, states and countries. However, the average values of the survey and the frequency of poor working time conditions would likely vary.

## Conclusion

The 14-item WTS is an effective tool for assessing working time exposures in a variety of full-time jobs with non-standard schedules. Furthermore, the two sub-scales differentiate between health and well-being consequences of extended and irregular work days and has important implications for future epidemiological investigations.

## Supplementary Information

**Additional file 1: Table A1.** Fit indices for exploratory factor analysis of the Work Time Scale in Department of Transportation (DOT) workers (*n*=174). **Table A2.** Fit indices for confirmatory factor analysis of the Work Time Scale in Department of Corrections (DOC) workers (*n*=114). **Table A3.** Fit indices for confirmatory factor analysis of the Work Time Scale in Manufacturing (MFG) workers (*n*=99). **Table A4.** Final Working Time Scale. Respondents were asked “Thinking about all jobs that you work, and including all overtime, say how often the following occurred over the LAST YEAR.” Respondents selected options on a 5-point Likert scale (1 = always, 2 = usually, 3 = sometimes, 4 = rarely, 5 = never).

## Data Availability

Due to the privacy of the participants, the dataset generated and analyzed during the study is not publicly available. The data that support the findings of this study are available from the corresponding author, [JMC], upon reasonable request.
