# Supplementary material for: The development and initial validation of a new working time scale for full-time workers with non-standard schedules
Source: BMC Public Health. 2022 Aug 20;22:1586. doi: 10.1186/s12889-022-13963-7 (PMC9392932; doi:10.1186/s12889-022-13963-7)
Supplement: Supplementary file 1 — Additional file 1: Table A1. Fit indices for exploratory factor analysis of the Work Time Scale in Department of Transportation (DOT) workers (n=174). Table A2. Fit indices for confirmatory factor analysis of the Work Time Scale in Department of Corrections (DOC) workers (n=114). Table A3. Fit indices for confirmatory factor analysis of the Work Time Scale in Manufacturing (MFG) workers (n=99). Table A4. Final Working Time Scale. Respondents were asked “Thinking about all jobs that you work, and including all overtime, say how often the following occurred over the LAST YEAR.” Respondents selected options on a 5-point Likert scale (1 = always, 2 = usually, 3 = sometimes, 4 = rarely, 5 = never). [file 12889_2022_13963_MOESM1_ESM.docx]

# **Appendix**

**Table A1. Fit indices for exploratory factor analysis of the Work Time Scale in Department of Transportation (DOT) workers (n=174)**

| **Model** | **Eigen Value** | **ꭓ^2^** | **df** | **RMSEA** | **CFI** | **TLI** | **SRMR** | **Model Comp** | **∆ꭓ^2^** | **∆df** |  |
| --- | --- | --- | --- | --- | --- | --- | --- | --- | --- | --- | --- |
|  |  |  |  |  |  |  |  |  |  |  |  |
| Work Time Scale | |  |  |  |  |  |  |  |  |  |  |
|  |  |  |  |  |  |  |  |  |  |  |  |
| 1 Factor | 8.515 | 460.614 | 77 | 0.172 | 0.801 | 0.765 | 0.073 |  |  |  |  |
|  |  |  |  |  |  |  |  |  |  |  |  |
| 2 Factors | 1.269 | 315.28 | 64 | 0.152 | 0.870 | 0.815 | 0.050 | 1-factor vs 2-factor | 145.33*** | 13 |  |
|  |  |  |  |  |  |  |  |  |  |  |  |
|  |  |  |  |  |  |  |  |  |  |  |  |
| 3 Factors | 0.913 | 151.634 | 52 | 0.106 | 0.948 | 0.910 | 0.031 | 2-factor vs 3-factor | 163.65*** | 12 |  |
|  |  |  |  |  |  |  |  |  |  |  |  |
| 4 Factors | 0.626 | 64.589 | 41 | 0.058 | 0.988 | 0.973 | 0.022 | 3-factor vs 4-factor | 87.05*** | 11 |  |
|  |  |  |  |  |  |  |  |  |  |  |  |
|  |  |  |  |  |  |  |  |  |  |  |  |

***P<.001

Model Comp=Model Comparison

**Table A2. Fit indices for confirmatory factor analysis of the Work Time Scale in Department of Corrections (DOC) workers (n=114)**

| **Model** | **Description** | **ꭓ^2^** | **df** | **RMSEA** | **CFI** | **TLI** | **SRMR** | **Model Comp** | **∆ꭓ^2^** | **∆df** |  |
| --- | --- | --- | --- | --- | --- | --- | --- | --- | --- | --- | --- |
|  |  |  |  |  |  |  |  |  |  |  |  |
| Work Time Scale | |  |  |  |  |  |  |  |  |  |  |
|  |  |  |  |  |  |  |  |  |  |  |  |
| 1 Factor | CFA with no modifications | 379.415 | 77 | 0.186 | 0.666 | 0.605 | 0.127 |  |  |  |  |
|  |  |  |  |  |  |  |  |  |  |  |  |
| 2 Factors | CFA with no modifications | 308.442 | 76 | 0.164 | 0.743 | 0.692 | 0.148 | 1-factor vs 2-factor | 70.973*** | 1 |  |
|  |  |  |  |  |  |  |  |  |  |  |  |
|  |  |  |  |  |  |  |  |  |  |  |  |
| 2 Factors (with mod) | CFA with modification indices (per Hinkin, 1998) | 148.758 | 70 | 0.099 | 0.913 | 0.887 | 0.076 | 2-factor vs 2-factor (with mod) | 159.684*** | 6 |  |
|  |  |  |  |  |  |  |  |  |  |  |  |
|  |  |  |  |  |  |  |  |  |  |  |  |

***P<.001

Model Comp=Model Comparison

**Table A3. Fit indices for confirmatory factor analysis of the Work Time Scale in Manufacturing (MFG) workers (n=99)**

| **Model** | **Description** | **ꭓ^2^** | **df** | **RMSEA** | **CFI** | **TLI** | **SRMR** | **Model Comp** | **∆ꭓ^2^** | **∆df** |
| --- | --- | --- | --- | --- | --- | --- | --- | --- | --- | --- |
|  |  |  |  |  |  |  |  |  |  |  |
| Work Time Scale | |  |  |  |  |  |  |  |  |  |
|  |  |  |  |  |  |  |  |  |  |  |
| 1 Factor | CFA with no modifications | 242.676 | 77 | 0.153 | 0.773 | 0.731 | 0.082 |  |  |  |
|  |  |  |  |  |  |  |  |  |  |  |
| 2 Factors | CFA with no modifications | 214.229 | 76 | 0.141 | 0.810 | 0.773 | 0.079 | 1-factor vs 2-factor | 28.447*** | 1 |
|  |  |  |  |  |  |  |  |  |  |  |
|  |  |  |  |  |  |  |  |  |  |  |
| 2 Factors (w/ mod) | CFA with modification indices (per Hinkin, 1998) | 136.321 | 70 | 0.101 | 0.909 | 0.882 | 0.073 | 2-factor vs 2-factor (w/ mod) | 77.908*** | 6 |
|  |  |  |  |  |  |  |  |  |  |  |
|  |  |  |  |  |  |  |  |  |  |  |

***P<.001

Model Comp=Model Comparison

**Table A4. Final Working Time Scale. Respondents were asked “Thinking about all jobs that you work, and including all overtime, say how often the following occurred over the LAST YEAR.” Respondents selected options on a 5-point Likert scale (1 = always, 2 = usually, 3 = sometimes, 4 = rarely, 5 = never).**

| **Items** | | **Domain** | **Factor** |
| --- | --- | --- | --- |
| Q1 | I worked more than 12 hours per day | Length | EIWD |
| Q2 | I worked more than 48 hours per week | Length | EIWD |
| Q3 | I worked overtime | Length | EIWD |
| Q4 | I worked some early morning hours between 5am and 8am | Time of day | EIWD |
| Q6 | I worked at least 3 evening hours after 6pm | Time of day | EIWD |
| Q7 | I worked at least 3 overnight hours between 11pm and 5am | Time of day | EIWD |
| Q8 | I worked 6 or more days in a row | Intensity | EIWD |
| Q11 | I was on call (expected to immediately provide work or service if contacted or called) | Control | LOC |
| Q12 | I worked mandatory overtime | Control | LOC |
| Q14 | I had to go to work unexpectedly at times when I was not scheduled to work | Predictability | LOC |
| Q15 | I unexpectedly had to work more than an hour later than I was scheduled to work | Predictability | LOC |
| Q18 | I worked on a Sunday | Free time | EIWD |
| Q19 | I worked on the weekend | Free time | EIWD |
| Q20 | I worked on a holiday | Free time | EIWD |

EIWD = Extended and irregular work days; LOC = lack of control
